# Effect of Weiss ring on peripapillary retinal nerve fiber layer thickness measurements using SD-OCT

**DOI:** 10.1038/s41598-022-22094-3

**Published:** 2022-10-17

**Authors:** Min-Su Kim, Ki-Yup Nam, Young Hoon Hwang, Min-Woo Lee, Woo-Hyuk Lee, Hyung-Bin Lim, Jung-Yeul Kim

**Affiliations:** 1 1.0 Eye Clinic, Daejeon, Republic of Korea; 2 Department of Ophthalmology, Chungnam National University College of Medicine, Daejeon, Republic of Korea; 3 Department of Ophthalmology, Chungnam National University College of Medicine, Sejong, Republic of Korea; 4 Department of Ophthalmology, Konyang University College of Medicine, Daejeon, Republic of Korea; 5 Department of Ophthalmology, Gyeongsang National University College of Medicine, Changwon, Republic of Korea

**Keywords:** Anatomy, Medical research

## Abstract

Spectral-domain optical coherence tomography (SD-OCT) must accurately identify and measure the peripapillary retinal nerve fiber layer (pRNFL) thickness to improve the repeatability and reproducibility, and reduce measurement errors. Because Weiss ring can be located in front of the optic disc, we hypothesized that it may affect pRNFL thickness measurements obtained using SD-OCT. We retrospectively reviewed the medical records of patients with (group W) and without (group N) Weiss ring, observed on OCT fundus image and an RNFL map devised using SD-OCT. Optic disc cube scans (200 × 200) were obtained to measure pRNFL thicknesses (superior, temporal, inferior, nasal, and average) at two consecutive visits. Pearson's correlation coefficient (*r*), intraclass correlation coefficient (ICC), and coefficient of variation (CV) were calculated. The *r* and ICC values for the pRNFL thickness measurements at the two visits were lower for group W compared to group N, but statistical significance was reached only for inferior pRNFL thickness. In addition, CV values were greater for group W compared to group N, but the differences were significant only for inferior and average pRNFL thickness measurements (*p* < 0.001 and *p* = 0.004, respectively). Weiss ring located near the optic disc can affect pRNFL thickness measurements and repeatability thereof, especially the inferior quadrant and average values. Therefore, it is important to identify the presence of Weiss ring when analyzing pRNFL thickness values.

## Introduction

Spectral-domain optical coherence tomography (SD-OCT) is widely used for the diagnosis of ophthalmic diseases^1–3^. SD-OCT detects structural changes in the retina, i.e., changes in the thickness of the central macula, macular ganglion cell-inner plexiform layer, and peripapillary retinal nerve fiber layer (pRNFL), which aids treatment decisions^4–7^. In glaucoma, ophthalmologists usually use numerical pRNFL thickness values obtained by SD-OCT to determine disease progression. Therefore, SD-OCT must accurately identify and measure the pRNFL thickness to improve the repeatability and reproducibility, and reduce measurement errors. Measurement errors in the pRNFL thickness using SD-OCT can be caused by low signal quality^8^, eye movements^9^, media opacity (e.g., cataracts)^10^, and vitreous opacity^11,12^. These factors make it difficult for the ophthalmologist to determine structural changes in the pRNFL using SD-OCT.

Weiss ring refers to a vitreous opacity or floater located near the optic disc, which is often incidentally found during a dilated fundus examination^13^. Weiss ring consists of peripapillary glial tissue attached to the posterior vitreous cortex, which develops after posterior vitreous detachment, and is used by retinal specialists to confirm the presence of complete posterior vitreous detachment. Because Weiss ring can be located in front of the optic disc, we hypothesized that it may affect pRNFL thickness measurements obtained using SD-OCT. In our previous study^14^, we found that inferior and average pRNFL were thinner in eyes with Weiss ring than in eyes without Weiss ring. However, since pRNFL thickness can vary from patient to patient, our colleagues thought that it would be more meaningful to analyze the agreement and difference of pRNFL thickness measurements at two consecutive visits in the same patients. To the best of our knowledge, no previous studies have quantitatively evaluated the effect of Weiss ring on the agreement and difference of pRNFL thickness measurements. The current study aimed to quantitatively analyze the effect of Weiss ring on the agreement and difference of pRNFL thickness measurements obtained using SD-OCT.

## Results

### Demographics

We included 71 eyes of 71 patients in this study (36 eyes in group W and 35 in group N). No significant differences were observed between the groups in age, sex, laterality, medical history, BCVA, IOP, SE, AL, or the interval between visits (*p* > 0.05) (Table 1).

**Table 1:** Baseline characteristics of the participants.

Characteristic	Weiss ring ( +) group	Weiss ring ( −) group	*p *value
Number of eyes	36	35	
Age (mean ± SD, y)	69.50 ± 6.94	70.23 ± 9.57	0.714*
Sex (male/female)	22/14	22/13	0.537^†^
Laterality (right/left eye)	19/17	18/17	0.549^†^
Diabetes mellitus (*n* [%])	9 (25.0%)	7 (20.0%)	0.413^†^
Hypertension (*n* [%])	13 (36.1%)	14 (40%)	0.463^†^
BCVA (mean ± SD, logMAR)	0.04 ± 0.11	0.09 ± 0.18	0.160*
Spherical equivalent (mean ± SD, diopters)	0.19 ± 1.62	− 0.20 ± 1.77	0.301*
Intraocular pressure (mean ± SD, mmHg)	15.69 ± 2.55	14.49 ± 2.76	0.060*
Axial length (mean ± SD, mm)	23.58 ± 1.07	23.49 ± 1.07	0.757*
Interval between visits (mean ± SD, months)	5.34 ± 4.18	6.18 ± 3.72	0.376*

*Comparison between groups 1 and 2 using the independent *t*-test.

^†^Comparison between groups 1 and 2 using the chi-square test.

*SD* standard deviation, *BCVA* best-corrected visual acuity, *logMAR* logarithm of the minimum angle of resolution.

### The location of Weiss ring at the first and second visits in group W

Weiss ring within the RNFL scan circle was observed in 25 and 29 eyes out of the 36 eyes in group W at the first and second visits, respectively. At the first visit, Weiss ring was mainly observed in the inferior quadrant (17/25, [68.0%]), followed by nasal (5/25, [20.0%]), temporal (2/25, [8.0%]), and superior (1/25, [4.0%]) quadrants. At the second visit, Weiss ring was mainly observed in the inferior quadrant (16/29, [55.2%]), followed by nasal (6/29, [20.7%]), superior (5/29, [17.2%]), and temporal (2/29, [6.9%]) quadrants (Table 2).

**Table 2:** Location of the Weiss ring at the first and second visits in group W.

Quadrants	First visit (n [%])	Second visit (n [%])
Superior	1 (4.0%)	5 (17.2%)
Temporal	2 (8.0%)	2 (6.9%)
Inferior	17 (68.0%)	16 (55.2%)
Nasal	5 (20.0%)	6 (20.7%)
Total	25 (100%)	29 (100%)

Among the 36 eyes in group W, 11 eyes at the first visit and 7 eyes at the second visit had Weiss ring outside the scan circle.

### Average and sectoral pRNFL thickness measurements

The average and sectoral pRNFL thickness measurements in both groups were not significantly different between the first and second visits (*p* > 0.05 for all) (Table 3).

**Table 3:** Comparisons of average and sectoral peripapillary retinal nerve fiber layer (pRNFL) thickness measurements at the first and second visits in groups W and N.

	Group W (Weiss ring [ +])			Group N (Weiss ring [ −])		
	First visit	Second visit	*p *value*	First visit	Second visit	*p* value*
Average pRNFL	95.36 ± 11.38	95.08 ± 12.17	0.788	98.34 ± 10.19	98.11 ± 10.69	0.457
Superior pRNFL	119.28 ± 15.73	116.83 ± 16.44	0.074	119.89 ± 15.77	119.03 ± 15.26	0.908
Temporal pRNFL	77.69 ± 15.15	79.58 ± 17.20	0.197	77.20 ± 11.75	77.11 ± 11.67	0.597
Inferior pRNFL	114.50 ± 24.27	114.50 ± 21.37	1.000	125.83 ± 18.43	125.29 ± 19.39	0.763
Nasal pRNFL	69.94 ± 12.15	69.33 ± 10.63	0.722	70.46 ± 10.67	70.77 ± 11.37	0.691

Data are presented as the mean ± standard deviation.

*p value for difference in RNFL measurements between the first and second visits using the paired *t*-test.

### Agreement in average and sectoral pRNFL thickness measurements between the first and second visits

**Figure 1:**
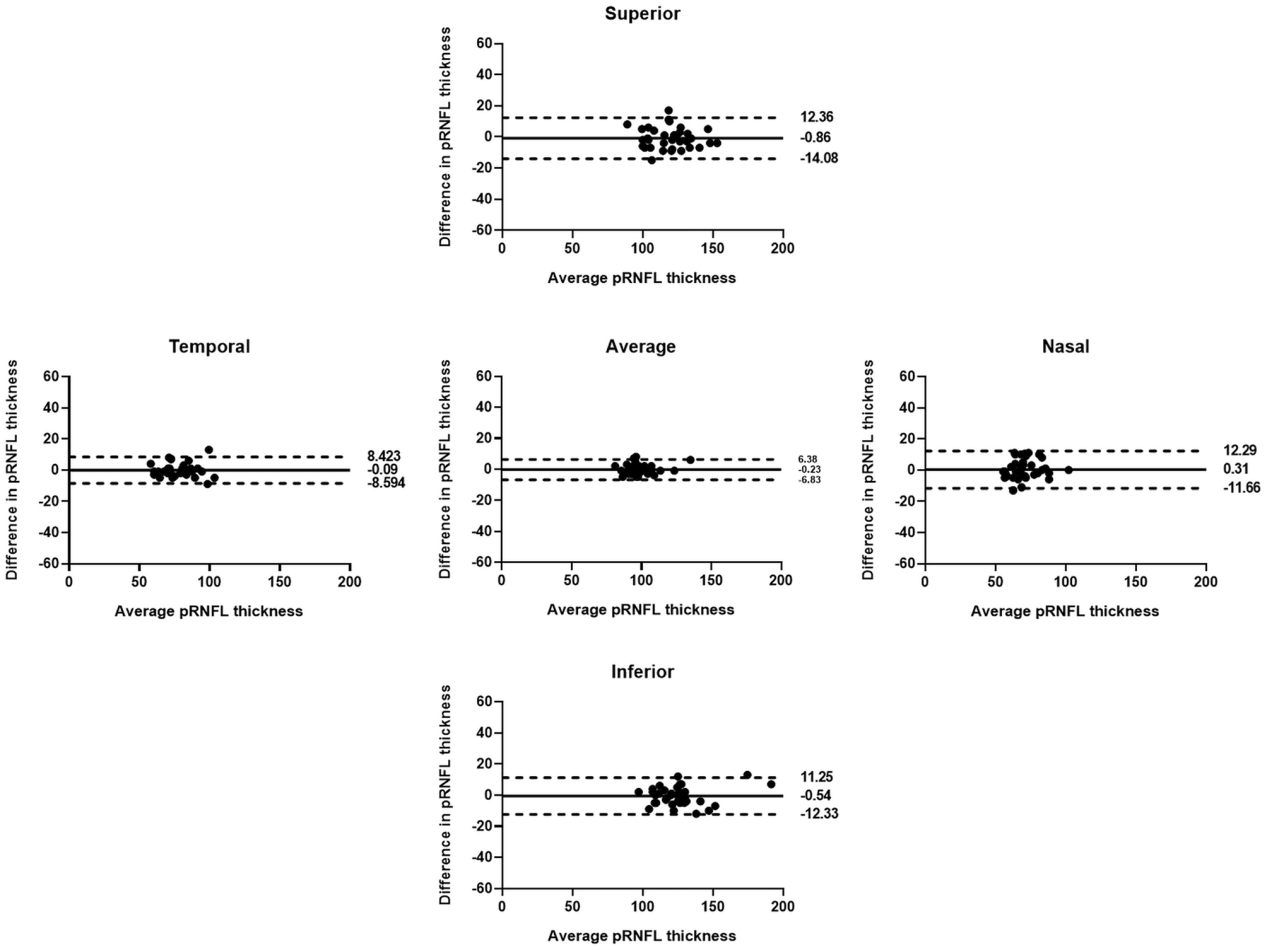
Bland–Altman plots showing the agreement of peripapillary retinal nerve fiber layer (pRNFL) thickness measurements at the two visits in group N. Solid lines represent bias (mean pRNFL thickness difference), and dotted lines represent the upper and lower limits of agreement. Mean bias and upper and lower limits of agreement are shown at the right end of each line. There were no differences in average or sectoral pRNFL thickness measurements between the visits, indicating good agreement.

**Figure 2:**
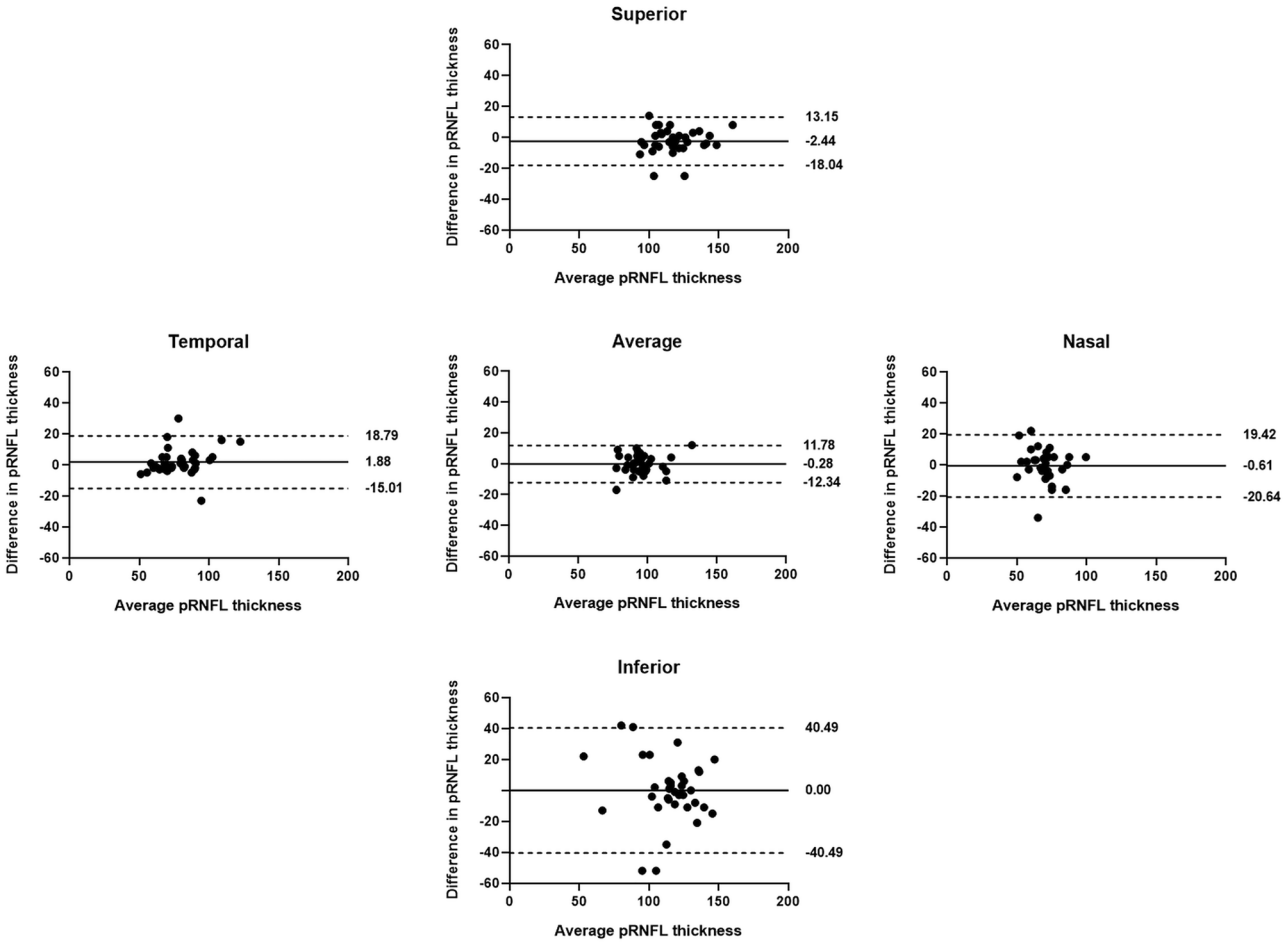
Bland–Altman plots for the agreement of peripapillary retinal nerve fiber layer (pRNFL) measurements between the two visits in group W. Solid lines represent bias (mean pRNFL thickness difference), and dotted lines represent the upper and lower limits of agreement. Mean bias and upper and lower limits of agreement are shown at the right end of each line. Differences in average and sectoral pRNFL thickness measurements between the visits were greater for group W compared to group N, especially for the inferior pRNFL thickness.

Table 4 shows the *r*, ICC, and CV values ​​for average and sectoral pRNFL thickness measurements at the first and second visits. A positive correlation, with statistically significant *r* and ICC values (*p* < 0.01), was observed for all values in both groups. The *r* and ICC values were lower for group W compared to group N, but the differences were only significant for inferior pRNFL thickness in group W (*r* = 0.597, 95% CI = 0.277–0.821; ICC = 0.744, 95% CI = 0.497–0.869) and group N (*r* = 0.951, 95% CI = 0.865–0.978; ICC = 0.974, 95% CI = 0.949–0.989). The CV values were greater for group W compared to group N, but the differences were significant only for the inferior and average pRNFL thickness measurements (*p* < 0.001 and *p* = 0.004, respectively). Similarly, the differences in average and sectoral pRNFL thickness measurements between the visits were greater for group W compared to group N, and were particularly pronounced for inferior pRNFL (Figs. 1 and 2).

**Table 4:** Pearson's correlation coefficient (*r*), intraclass correlation coefficient (ICC), and coefficient of variation (CV) for average and sectoral peripapillary retinal nerve fiber layer (pRNFL) thickness measurements at the first and second visits in groups W and N.

	Group W (Weiss ring [ +])				Group N (Weiss ring [ −])			*p *value^†^
	*r* (95% CI)*	ICC (95% CI)*	CV		*r* (95% CI)*	ICC (95% CI)*	CV	
Average pRNFL	0.866 (0.752–0.927)	0.927 (0.857–0.963)	3.64		0.949 (0.836–0.979)	0.973 (0.947–0.987)	1.99	**0.004**
Superior pRNFL	0.879 (0.745–0.950)	0.935 (0.872–0.957)	3.77		0.906 (0.810–0.957)	0.950 (0.902–0.975)	3.31	0.537
Temporal pRNFL	0.865 (0.713–0.967)	0.924 (0.851–0.961)	4.93		0.931 (0.894–0.969)	0.964 (0.930–0.982)	2.86	0.052
Inferior pRNFL	0.597 (0.277–0.821)	0.744 (0.497–0.869)	10.15		0.951 (0.865–0.978)	0.974 (0.949–0.987)	2.66	** < 0.001**
Nasal pRNFL	0.604 (0.262–0.829)	0.749 (0.508–0.872)	7.75		0.848 (0.695–0.922)	0.917(0.835–0.958)	4.91	0.062

*All *p *values < 0.01 for Pearson’s correlation coefficient (r) and ICC.

^†^*p* value for the difference in CV between groups W and N.

*CI* confidence interval.

Significant values are in bold.

### Differences in average and sectoral pRNFL thickness measurements between the first and second visit

Differences in pRNFL thickness between the first and second visits were greater for group W compared to group N. These differences were particularly significant for inferior and average pRNFL thickness measurements (*p* < 0.001 and *p* = 0.004, respectively) (Table 5).

**Table 5:** Comparisons of differences in average and sectoral peripapillary retinal nerve fiber layer (pRNFL) thickness at the first and second visits.

	Differences in pRNFL thickness measurements between first and second visits (mean ± SD, 95% CI)		
	Group W (Weiss ring [ +])	Group N (Weiss ring [ −])	*p *value*
Average pRNFL	4.83 ± 3.73 (3.65–6.10)	2.74 ± 1.92 (2.12–3.42)	**0.004**
Superior pRNFL	6.06 ± 5.64 (4.32–8.05)	5.43 ± 3.99 (4.13–6.83)	0.591
Temporal pRNFL	5.61 ± 6.75 (3.67–7.97)	3.17 ± 2.92 (2.29–4.24)	0.053
Inferior pRNFL	14.56 ± 14.45 (10.14–19.63)	4.83 ± 3.54 (3.71–6.00)	** < 0.001**
Nasal pRNFL	7.33 ± 7.04 (5.25–9.91)	4.77 ± 3.74 (3.60–6.00)	0.060

*SD* standard deviation, *CI* confidence interval.

*p value for the differences in pRNFL thickness measurements between groups W and N using the independent *t*-test.

Significant values are in bold.

### Relationships of differences in average and inferior pRNFL thickness measurements with clinical factors

Simple regression analyses were performed for the relationships between differences in pRNFL thickness measurements (average and inferior) and clinical factors in group W (Table 6). Only diabetes (dummy variables: 1 for presence and 0 for absence) was positively correlated with differences in average (β ± standard deviation [SD] = 4.370 ± 3.741, *p* = 0.001) and inferior (β ± SD = 13.333 ± 11.222, *p* = 0.014) pRNFL thickness measurements. Therefore, the differences in average and inferior pRNFL thickness measurements in group W were greater for diabetics compared to non-diabetics. Because none of the other clinical factors had a statistically significant correlation with thickness, multiple regression analysis was not performed.

**Table 6:** Simple regression analysis of the relationships between clinical factors and differences in average and inferior pRNFL thickness measurements at the first and second visits in group W.

	Simple regression analysis (β ± SD)			
	Average	*p *value*	Inferior	*p *value†
Age (y)	–0.016 ± 5.961	0.863	–0.038 ± 17.195	0.916
Sex (M = 0, F = 1)	1.909 ± 4.091	0.136	1.195 ± 14.091	0.813
Laterality (R = 0, L = 1)	–1.245 ± 5.421	0.325	0.285 ± 14.421	0.954
Diabetes mellitus ([ +] = 1, [–] = 0)	4.370 ± 3.741	**0.001**	13.333 ± 11.222	**0.014**
Hypertension ([ +] = 1, [–] = 0)	2.187 ± 4.043	0.091	9.003 ± 11.304	0.072
Lens status (phakic = 0, pseudophakic = 1)	1.273 ± 4.727	0.579	0.848 ± 14.485	0.924
Best-corrected visual acuity	2.680 ± 4.820	0.656	8.184 ± 14.509	0.727
Spherical equivalent (Diopter)	0.298 ± 4.778	0.451	1.433 ± 14.290	0.348
Intraocular pressure (mmHg)	–0.017 ± 5.098	0.947	–0.074 ± 15.720	0.940
Axial length (mm)	–0.391 ± 13.961	0.586	–0.897 ± 35.108	0.750
Interval between visits (months)	0.018 ± 4.737	0.907	–0.302 ± 16.168	0.612

*p value for the simple regression analysis between differences in average pRNFL thickness measurements and clinical factors.

^†^*p *value for the simple regression analysis between differences in inferior pRNFL thickness measurements and clinical factors.

Significant values are in bold.

## Discussion

In the current study, the r and ICC values for pRNFL thickness measured at two consecutive visits were lower for eyes with Weiss ring compared to those without Weiss ring. In particular, there was a significant difference in inferior pRNFL thickness. Conversely, CV values were greater for eyes with Weiss ring compared to those without Weiss ring, suggesting significant differences in inferior and average pRNFL thickness measurements. In addition, the differences were greater for eyes with Weiss ring compared to those without Weiss ring, with significant results for inferior and average pRNFL thickness measurements. These results suggest that Weiss ring mainly affects the inferior pRNFL thickness measurements on SD-OCT, which may also affect the average pRNFL thickness measurement.

**Figure 3:**
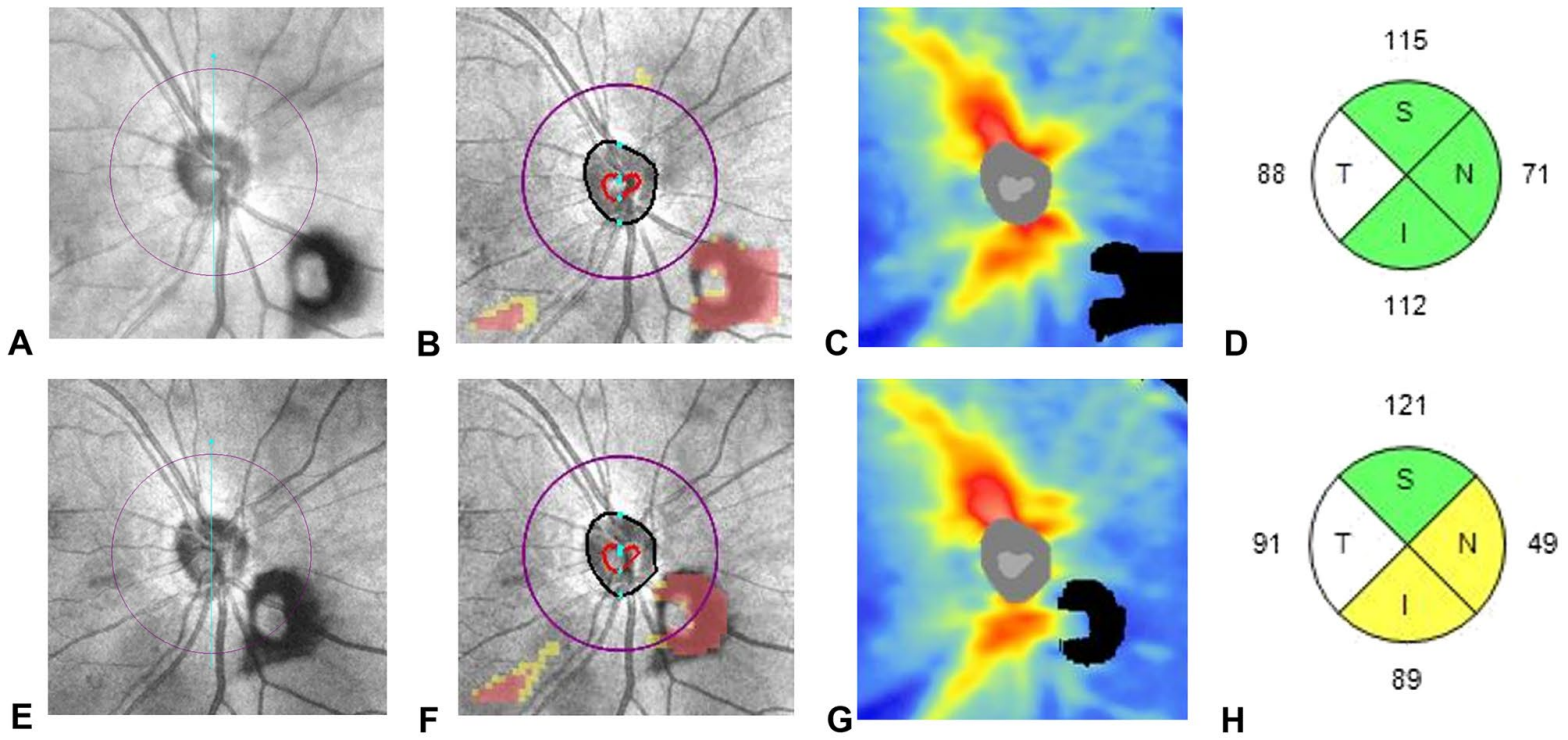
A representative SD-OCT scan (optic disc cube: 200 × 200) for group W eyes. A 64-year-old male patient presented with a right eye symptomatic floater. On dilated fundus examination, a Weiss ring was observed near the optic disc, without any peripheral retinal break (**A**–**D**). OCT fundus image showing a Weiss ring in the inferonasal area of ​​the optic disc (**A**). In the deviation map, a red cluster is observed outside the 3.46-mm-diameter scan circle (purple circle) from the center of the optic disc, in the same location as the Weiss ring in the OCT fundus image (**B**). In the thickness map, a black defect is observed in the same location as the Weiss ring in the OCT fundus image (**C**). Quadrant map showing the pRNFL thickness. After 4 months, the follow-up SD-OCT image showed a Weiss ring within the scan circle (**E**–**G**). Quadrant map showing reduced inferior and nasal pRNFL thicknesses compared to the previous map (**H**).

Weiss ring is a type of vitreous floaters commonly found near the fundus^13^; ophthalmologists can easily observe movement of the Weiss ring in front of the optic disc during fundus examination. Several case reports have documented the effect of vitreous floaters on pRNFL thickness measurements^11,12^. In the aforementioned studies, vitreous floaters appeared as a black defect in the pRNFL thickness map, red cluster in the pRNFL deviation map, and black shadow in the B-scan extracted from the pRNFL scan circle. Therefore, vitreous floaters may affect the temporal-superior-nasal-inferior-temporal (TSNIT) profiles, quadrant maps, and clock-hour maps, resulting in errors in pRNFL thickness measurements. In the present study, the Weiss ring also appeared as a black defect in the pRNFL thickness map and red cluster in the pRFNL deviation map, which may cause measurement errors in the pRNFL thickness (Fig. 3).

In the current study, the Weiss ring mainly affected the agreement/differences in inferior pRNFL thickness measurements, probably because more than half of the Weiss rings were present in the inferior quadrant at the first and second visits. We speculate that Weiss ring formed mostly in the inferior quadrant of the optic disc due to the effects of gravity after complete posterior vitreous detachment. Clinically, the inferior pRNFL thickness is important for the diagnosis and progression of glaucoma. Therefore, ophthalmologists may make errors in the diagnosis of glaucoma in patients with Weiss ring.

Because the differences in pRNFL thickness measurements between the groups were only significant for inferior and average pRNFL values, regression analysis was performed only for these measurements. In group W, diabetes correlated with the differences in inferior and average pRNFL thickness measurements. Foos et al.^15^ reported precocious liquefaction of the vitreous in diabetic patients. Sebag et al.^16^ reported abnormal collagen cross-linking and non-enzymatic glycation of vitreous in diabetic patients, suggesting that these changes to the vitreous may play an important role in the progression of proliferative diabetic retinopathy. Instability of the vitreous in diabetic patients due to the aforementioned reasons may allow greater movement of the Weiss ring. However, further research is needed to confirm this.

This study had several limitations. First, considering the frequency of Weiss ring occurrence in the general population, it included a small number of patients over a relatively short period, thereby predisposing it to selection bias. The small sample size and short study duration were due to the difficulty in long-term follow-up of patients with floaters in clinical practice. Second, the possibility of measurement error due to differences in the placement of the calculation circle cannot be excluded. Differences in the calculation circle may affect pRNFL thickness measurements. Additional studies are needed to analyze the pRNFL thickness after matching the scan locations in successive examinations using software such as guided progression analysis in Cirrus HD-OCT. Third, the Weiss ring can cause segmentation errors in the B-scan, which may lead to measurement errors in the pRNFL thickness. In this study, additional manipulations were not performed, and the numerical information of quadrant maps was used as written. It is very important to measure the accurate pRNFL thickness by correcting the segmentation errors in b-scan. However, our study simulated real-world clinical practice. Therefore, it can also be meaningful that in eyes with Weiss ring, the only numerical values indicated in the pRNFL thickness map (quadrant maps and clock-hour maps) should not be interpreted as written. Further study is needed to analyze the pRNFL thickness measurements by correcting the segmentation errors. Finally, we did not account for the decrease in pRNFL thickness due to aging. However, the average interval between visits was about 6 months, and no significant association was observed for the interval between visits and differences in pRNFL thickness in regression analysis. Therefore, age-related decline in pRNFL thickness probably did not affect the study results. Despite these limitations, our study had several strengths. It was the first study to quantitatively analyze the agreement in average and sectoral pRNFL thickness measurements in eyes with and without Weiss ring. In addition, we compared differences in pRNFL thickness measurements between eyes with and without Weiss ring, and analyzed their relationships with clinical factors.

In conclusion, when patients are examined, pRNFL thickness measurements may be inaccurate if only the numerical information from the quadrant and clock-hour maps or TSNIT profile are used, without considering the presence of the Weiss ring. Therefore, when changes in pRNFL thickness are determined using the optic disc cube scan in SD-OCT, it is important to exclude abnormalities in the deviation (red clusters) and thickness (black defect) maps, as well as Weiss ring.

## Methods

### Participants

This retrospective, case–control study was approved by the Institutional Review Board of Chungnam National University Hospital, Republic of Korea (no.: 2021-03-074). The requirement for obtaining informed consent was waived due to the retrospective nature of the study. The study protocol adhered to the Declaration of Helsinki.

We retrospectively analyzed the medical records of patients who presented to the retinal clinic of Chungnam National University Hospital between January 2017 and December 2020 with symptomatic floaters. We collected information on age, sex, laterality, medical history (e.g., diabetes and hypertension), lens status, concomitant retinal disease, previous ocular surgery, intervals between visits, best-corrected visual acuity (BCVA), spherical equivalent (SE), intraocular pressure (IOP; CT-80; Topcon Corp., Tokyo, Japan), and axial length (AL; IOL Master; Carl Zeiss Meditec, Dublin, CA, USA), as well as findings of the dilated fundus examination. We included patients with a history of uncomplicated cataract surgery or laser treatment for peripheral retinal break or degeneration.

Patients with optic nerve diseases, such as glaucoma, optic atrophy, or optic neuritis, which may affect pRNFL thickness, were excluded. In addition, patients with diabetic or hypertensive retinopathy, IOP > 21 mmHg, high myopia (SE <  − 6.5 D or AL > 26.5 mm), macular disease (e.g., age-related macular degeneration, epiretinal membrane, or macular hole), retinal vascular disease (e.g., retinal vein or artery occlusion), previous uveitis, previous pars plana vitrectomy, or an interval between visits > 1 year were also excluded.

The included eyes were divided into two groups. Group W included eyes that had Weiss ring clearly visible on the OCT fundus image; red clusters and black defects visible on the RNFL deviation and thickness maps (optic disc cube: 200 × 200) in the same location as the Weiss ring seen on the OCT fundus image; and the Weiss ring defect observed at least once during two consecutive visits, within a 3.46-mm-diameter RNFL scan circle (purple circle) from the center of the optic disc on the deviation map (Fig. 3). Group N included eyes with no Weiss ring on the OCT fundus image, with or without symptomatic floaters. Group N and W eyes were matched (ratio, 1:1) on the basis of age and AL. If both eyes of a participant fulfilled the inclusion criteria, one was randomly selected for inclusion.

### Determination of the location of Weiss ring

In group W, the location of Weiss ring on OCT fundus images taken at the first and second visits were divided into superior, inferior, nasal, and temporal quadrants. If the Weiss ring was visible in two quadrants, two observers (M.S.K. and H.B.L.) checked the OCT fundus image and determined the location of Weiss ring. An experienced adjudicator (J.Y.K.) settled disagreements between the two observers.

### Peripapillary RNFL thickness measurement using the quadrant map

One experienced examiner performed SD-OCT scans (optic disc cube: 200 × 200) using the Cirrus HD-OCT 5000 (Carl Zeiss Meditec). The pRNFL thickness was calculated for four quadrants (superior, temporal, inferior, and nasal) within the scan circle, ​​and the average total pRNFL thickness was calculated from the optic disc cube (200 × 200 scan). Images with signal strength ≥ 8 were included in this study.

### Statistical analyses

The statistical analyses were performed using SPSS software (version 23.0; IBM Corp., Armonk, NY, USA). The chi-square test was used to compare sex, laterality, and medical history between groups W and N. The independent *t*-test was used to compare the BCVA, SE, IOP, AL, interval between visits, and absolute differences in pRNFL thicknesses (average, superior, temporal, inferior, and nasal) between the first and second visits. The paired *t*-test was used to compare the pRNFL thickness measurements between the first and second visits in groups W and N. Pearson’s correlation coefficient (*r*), intraclass correlation coefficient (ICC), and coefficient of variation (CV) values were calculated to evaluate the degree of agreement between measurements. The *r* and ICC values for groups W and N were manually compared using 95% confidence intervals (CIs), and CV values for groups W and N were compared using the independent *t*-test. In addition, Bland–Altman plots were produced to show differences between groups W and N. Simple regression analyses were performed to determine the relationships between absolute differences in pRNFL thicknesses and clinical factors. Statistical significance was set at *p* < 0.05.

## Data Availability

The datasets generated and analysed during the current study are available from the corresponding author on reasonable request.

## References

[1] Murthy RK, Haji S, Sambhav K, Grover S, Chalam KV (2016). Clinical applications of spectral domain optical coherence tomography in retinal diseases. Biomed. J..

[2] Chen TC (2018). Spectral-domain OCT: helping the clinician diagnose glaucoma: a report by the American academy of ophthalmology. Ophthalmology.

[3] Golbaz I (2011). Quantification of the therapeutic response of intraretinal, subretinal, and subpigment epithelial compartments in exudative AMD during anti-VEGF therapy. Invest. Ophthalmol. Vis. Sci..

[4] Mori S, Hangai M, Sakamoto A, Yoshimura N (2010). Spectral-domain optical coherence tomography measurement of macular volume for diagnosing glaucoma. J. Glaucoma.

[5] Nakatani Y, Higashide T, Ohkubo S, Takeda H, Sugiyama K (2011). Evaluation of macular thickness and peripapillary retinal nerve fiber layer thickness for detection of early glaucoma using spectral domain optical coherence tomography. J. Glaucoma..

[6] Hwang HS, Chae JB, Kim JY, Kim DY (2017). Association between hyperreflective dots on spectral-domain optical coherence tomography in macular edema and response to treatment. Invest. Ophthalmol. Vis Sci..

[7] Kriechbaum K (2009). Association of retinal sensitivity and morphology during antiangiogenic treatment of retinal vein occlusion over one year. Ophthalmology.

[8] Balasubramanian M, Bowd C, Vizzeri G, Weinreb RN, Zangwill LM (2009). Effect of image quality on tissue thickness measurements obtained with spectral domain-optical coherence tomography. Opt. Express..

[9] Langenegger SJ, Funk J, Töteberg-Harms M (2011). Reproducibility of retinal nerve fiber layer thickness measurements using the eye tracker and the retest function of Spectralis SD-OCT in glaucomatous and healthy control eyes. Invest. Ophthalmol. Vis. Sci..

[10] Lee DW, Kim JM, Park KH, Choi CY, Cho JG (2010). Effect of media opacity on retinal nerve fiber layer thickness measurements by optical coherence tomography. J. Ophthalmic Vis. Res..

[11] Hwang YH, Kim YY (2012). Effect of peripapillary vitreous opacity on retinal nerve fiber layer thickness measurement using optical coherence tomography. Arch. Ophthalmol..

[12] Hardin JS, Taibbi G, Nelson SC, Chao D, Vizzeri G (2015). Factors affecting cirrus-HD OCT Optic disc scan quality: a review with case examples. J. Ophthalmol..

[13] Akiba J, Ishiko S, Yoshida A (2001). Variations of Weiss's ring. Retina.

[14] Lee MW (2022). The weiss ring, a major confounding factor for measurements of peripapillary retinal nerve fiber layer thickness. Am. J. Ophthalmol..

[15] Foos RY, Kreiger AE, Forsythe AB, Zakka KA (1980). Posterior vitreous detachment in diabetic subjects. Ophthalmology.

[16] Sebag J, Buckingham B, Charles MA, Reiser K (1992). Biochemical abnormalities in vitreous of humans with proliferative diabetic retinopathy. Arch. Ophthalmol..

